# Process optimization and antioxidative activity of polyphenols derived from different seaweed species *Sargassum Miyabei*, *Undaria Pinnatifida Suringar*, and *Sargassum Thunbergii*

**DOI:** 10.1002/fsn3.2818

**Published:** 2022-05-11

**Authors:** Shan He, Yang Zhang, Yang Yuan, Muhammad Adil Farooq, Muhammad Shoaib Fayyaz, Dongxiao Su, Qinzhu Zeng, Abdul Rahaman

**Affiliations:** ^1^ 47875 School of Chemistry and Chemical Engineering Guangzhou University Guangzhou China; ^2^ Institute for NanoScale Science and Technology College of Science and Engineering Flinders University Bedford Park Australia; ^3^ Department of Food Science & Technology Khwaja Fareed University of Engineering & Information Technology Rahim Yar Khan Pakistan; ^4^ 26467 School of Food Science and Engineering South China University of Technology Guangzhou China

**Keywords:** chromatography–mass spectrometry (GC‐MS), polyphenols, seaweed, antioxidation, process optimization

## Abstract

The aim of this study was to extract the polyphenols from three major seaweed species such as *Sargassum miyabei, Undaria pinnatifida suringar*, and *Sargassum thunbergii,* which are found in the coastal province (Guangdong), a longest coastal line in China. It was found that the *Sargassum thunbergii* produced more polyphenols (34.99 mg) as compared to *Sargassum miyabei* (23.26 mg) and *Undaria pinnatifida suringar* (25.34 mg), respectively. The orthogonal method was used for the extraction of phenolic compounds and extraction condition of each seaweed species was optimized. The antioxidant activity of extracted polyphenols from all three species stated that the polyphenols extracted from *Undaria pinnatifida suringar* demonstrated the highest antioxidative activity. Furthermore, gas chromatography–mass spectrometry (GC‐MS) was used for qualitative analysis of polyphenols, which revealed that the major components of polyphenols extracted from *Undaria pinnatifida suringar* were gallic acid and arbutin followed by syringate in *Sargassum miyabei* and phloretin *in Sargassum thunbergii*.

## Graphical abstract

The aim of this study was to extract the polyphenols from three major seaweed species, Sargassum miyabei, Undaria pinnatifida suringar, and Sargassum thunbergii, which are found in the coastal province (Guangdong), a longest coastal line in China. The orthogonal method was used for extraction and condition of each seaweed species was optimized.

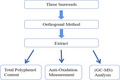

## 1 INTRODUCTION

Commercially available varieties of marine macroalgae are commonly referred to as seaweeds. Seaweeds serve as an important source of bioactive natural substances such as polyphenolic compounds, carotenoid pigments, and fucoidants due to their valuable antioxidant, anticoagulant, antibiotic, and antiulcer activities. There has been an increasing interest in the past few years in the extraction of polyphenolic compounds from seaweeds. Wang et al. (2009) applied 12 seaweed species isolated from Iceland as the raw material for the extraction of polyphenolic compounds and reported that the total phenolic content of these 12 seaweed species varied greatly, i.e., from 2% to 25%. Rajauria et al. (2016) extracted polyphenolic compounds from the Irish seaweed *Himanthalia elongate* followed by the separation of the crude extract by applying various techniques. They stated that the phenolic contents varied from 5.25% to 31% as separated by liquid–liquid partition‐column chromatography‐methanol subfraction and liquid–liquid partition‐ethyl acetate fraction, respectively. China and Indonesia are the largest seaweed producers with over 23 million tons of aggregated seaweed production in the world. Each produced more than 10 million tons of seaweed in 2017 (Buschmann et al., 2017). Among them, Guangdong Province is a major hub of seafood production including seaweed cultivation and production. It is the longest coastline (8500 km) covering all Chinese provinces, and approximately a fifth of the seafood production (over 180,000 tons in 2017). However, the efficiency of polyphenolic compound extraction from the major seaweed species of Guangdong Province has not yet been comprehensively studied and compared.

The antioxidative activity of polyphenolic compounds extracted from seaweed has been broadly studied. In this regard, Kajal et al. (2015) used different in vitro systems (DPPH, ABTS, HO radical scavenging activities, H_2_O_2_ scavenging ability, and Fe^2+^ ion chelating ability) to evaluate the antioxidative activity of three seaweeds (*Hypnea musciformis*, *H*. *valuentiae*, and *Jania rubens*) collected from the Gulf of Mannar on the southeastern coast of India. They indicated that *H*. *valuentiae* was the best specie among the three seaweeds due to its higher antioxidant activity and could be used as a potential food preservative. Shipeng et al. (2015) applied a more advanced technique, supercritical carbon dioxide extraction, to produce seaweed oil from the *Sargassum horneri* seaweed and reported that the extracted oil had a significant correlation between the antioxidative activity and polyphenolic content. The extracted seaweed oil with the highest antioxidative activity (68.38% measured by the DPPH method and 83.51% measured by the ABTS method) also contained the highest total polyphenolic content (0.64 mg/g). However, breakdown studies regarding the composition of each polyphenolic compound in the extracted polyphenolic complex and the relationship between this composition and the antioxidative activity of the polyphenolic complex have rarely been previously conducted.

Among the various seaweeds found along the coastline of Guangdong Province, *Sargassum miyabei, Undaria pinnatifida suringar,* and *Sargassum thunbergii* are the most abundantly available irrespective of the season (Shipeng et al., 2015). Although the antioxidative activity of polyphenolic compounds extracted from seaweeds has been proven by many previous studies, there is scant information regarding the antioxidative effects of the polyphenolic compounds extracted from these species, and from this crucial costal region of China. The results from this study will fulfill this knowledge gap. Furthermore, the seaweed specie with the best efficiency of polyphenolic compounds extraction and the polyphenols contributing the most toward antioxidative activity can be found. Therefore, this study will provide valuable information for the development of regional seaweed production in Guangdong Province, China, from both scientific and industrial points of view.

## 2 MATERIALS AND METHODS

### 2.1 Materials

The three seaweed species *Sargassum miyabei*, *Undaria pinnatifida suringar*, and *Sargassum thunbergii* were collected from the ocean region close to the coastal line of Guangdong Province, China. All chemicals were purchased from Sigma‐Aldrich Corporation.

### 2.2 Processing optimization of polyphenol complexes extracted from seaweeds

Extraction time, extraction temperature, concentration of ethanol, and the ratio of seaweed to extraction liquid were considered as four key factors for optimization. Each factor was tested at three levels (Table 1). The experiments were carried out according to the orthogonal design method. Five grams of each dried seaweed was mixed with ethanol in designed ratios (1:8, 1:9, and 1:10, Table 1). Three different concentrations (70%, 80%, and 90%) of ethanol were set for the trials (Table 1). The mixture of dried seaweed powder and ethanol was placed in a water bath and sonicated for the designed time (3 h, 4 h, and 5 h) and temperature (50°C, 60°C, and 70°C) (Table 1), then filtered with suction on No. 617 paper. The volume of the permeates collected from the filtration was measured. A total of nine trials (Table 2) with different processing conditions were formed according to the orthogonal design.

**TABLE 1:** Levels of key factors in the extraction of polyphenol complex

Factor	Level		
	1	2	3
Processing temperature (°C)	50	60	70
Ethanol concentration (%)	70	80	90
Ratio of powder to liquid	1:8	1:9	1:10
Processing time (h)	3	4	5

**TABLE 2:** Orthogonal design experiment results and analysis

Trial	Factors				Total polyphenols content^1^ (mg)		
	Processing Temperature (°C) (A)	Ethanol concentration (min) (B)	Ratio of powder to liquid (C)	Processing time (h) (D)	*Sargassum miyabei*	*Undaria pinnatifida suringar*	*Sargassum thunbergii*
1	1	1	1	1	11.05^a^ ± 1.21	15.32^b^ ± 0.63	15.7^b^ ± 0.62
2	1	2	2	2	9.52^c^ ± 0.25	10.74^a^ ± 1.36	16.75^b^ ± 0.85
3	1	3	3	3	5.24^d^ ± 0.23	6.78^d^ ± 0.52	10.43^a^ ± 1.23
4	2	1	2	3	14.72^b^ ± 0.56	11.75^a^ ± 0.32	8.77^c^ ± 0.63
5	2	2	3	1	17.97^b^ ± 1.96	11.54^a^ ± 0.85	10.79^a^ ± 0.86
6	2	3	1	2	8.35^c^ ± 1.03	7.49^c^ ± 0.21	6.30^d^ ± 0.62
7	3	1	3	2	14.79^b^ ± 0.89	16.50^b^ ± 1.03	25.67^e^ ± 1.63
8	3	2	1	3	11.64^a^ ± 0.62	13.93^b^ ± 0.42	21.36^f^ ± 1.20
9	3	3	2	1	11.30^a^ ± 0.45	5.45^d^ ± 0.85	14.55^b^ ± 0.45
^2^K_1sm_	0.8567	1.3467	1.0300	1.3400			
^2^K_2sm_	1.3633	1.3000	1.1833	1.0833			
^2^K_3sm_	1.2533	1.8267	1.2600	1.0500			
^3^R_sm_	0.5067 (R)	0.5200 (R)	0.2300	0.2900	B > A > D > C		
OPC_sm_					A_2_B_1_C_3_D_1_		
^2^K_1ups_	1.0900	1.4500	1.2200	1.0733			
^2^K_2ups_	1.0200	1.2033	0.9267	1.1533			
^2^K_3ups_	1.1933	0.6500	1.1567	1.0767			
^3^R_ups_	0.1733	0.8000	0.2933	0.0800		B > C > A > D	
OPC_ups_						A_3_B_1_C_1_D_2_	
^2^K_1st_	1.4267	1.6667	1.4433	1.3633			
^2^K_2st_	0.8567	1.6233	1.3300	1.6200			
^2^K_3st_	2.0467	1.0400	1.5567	1.3467			
^3^R_st_	1.1900	0.6267	0.2267	0.2733			A > B > D > C
OPC_st_							A_3_B_1_C_1_D_2_
Process by OPC					23.26^e^ ± 1.62	25.34^e^ ± 0.65	34.99^g^ ± 0.98

Note

Among each trial, different superscripts indicate a significant difference (*p* < .05) according to the one‐way ANOVA and LSD test.

OPC, Optimized processing condition; OPC_sm_, Optimized processing condition for *Sargassum miyabei*; OPC_st_, Optimized processing condition for *Sargassum thunbergii*; OPC_ups_, Optimized processing condition for *Undaria pinnatifida suringar*.

^1^ Average of three readings per trial ± standard deviation.

^2^ K_1_, K_2_, and K_3_ indicate the sum of the sensory scores corresponding to level 1, level 2, and level 3. K_sm_: K value of *Sargassum miyabei*; K_1ups_: K value of *Undaria pinnatifida suringar*; K_st_: K value of *Sargassum thunbergii*.

^3^ R = MaxK_i_–MinK_i_ (i = 1, 2 or 3). R_sm_: R value of *Sargassum miyabei*; R_ups_: R value of *Undaria pinnatifida suringar*; R_st_: R value of *Sargassum thunbergii*.

### 2.3 Determination of total polyphenol content

The total polyphenol content of the permeate after filtration was determined in accordance with the Folin–Ciocalteu method described by Kajal et al. (2015) with minor modifications. An aliquot of 1 ml of each permeates after filtration prepared from each of the nine trials was mixed with 5 ml of the Folin–Ciocalteu reagent (10% in distilled water) in a test tube. After 5 min, 4 ml of sodium carbonate (7.5% in distilled water) was added to each tube before the test tubes were cap screwed and vortexed. The samples were incubated for 2 h at room temperature in darkness. The absorbance was measured at 725 nm with a UV–vis spectrophotometer (Ultrospec 3000 pro, Amersham Pharmacia Biotech, Ltd.). A standard curve with serial phloroglucinol solutions (ranging from 20 lg/ml to 100 lg/ml) was used for calibration. The total polyphenol content in the permeate after filtration was calculated as the polyphenol content of 1 ml aliquot times the volume ratio of permeate after filtration (Table 3).

**TABLE 3:** Antioxidative activity of polyphenol complex produced from optimized processing conditions of *Sargassum miyabei, Undaria pinnatifida suringar*, and *Sargassum thunbergii*

Polyphenols extracted from seaweed species	Antioxidative activity^1^ (%)		
	DPPH radical scavenging activity measurement	Hydroxyl radical scavenging activity measurement	Lipid peroxidation inhibition measurement
*Sargassum miyabei*	42.83^a^ ± 2.36	43.82^a^ ± 1.34	52.35^a^ ± 2.36
*Undaria pinnatifida suringar*	92.95^b^ ± 5.21	72.61^b^ ± 5.32	78.97^b^ ± 5.63
*Sargassum thunbergii*	39.31^c^ ± 1.63	34.29^c^ ± 1.75	30.93^c^ ± 2.45

Note

For each trial, the different superscripts in the same column indicate a significant difference (*p* < .05) according to the one‐way ANOVA and LSD test.

^1^ Average of three readings per trial ± standard deviation.

### 2.4 Antioxidation measurement

Three measurements, DPPH radical scavenging activity, hydroxyl radical scavenging activity, and lipid peroxidation, were utilized to investigate antioxidative activity. The three permeates after filtration produced from optimized conditions were freeze dried to powder, then redissolved in 70% ethanol at a ratio of 10% (w/v). The redissolved solutions were used for antioxidation measurements.

#### 2.4.1 DPPH radical scavenging activity measurement

The DPPH radical scavenging activity method was utilized to investigate antioxidative activity. The DPPH radical scavenging activity of the freeze‐dried powders was measured according to the work published by Xie and Schaich (2014) with a slight modification. A total of 2 ml of the redissolved solutions was mixed with 0.2 ml of DPPH solution (0.4 mM in ethanol), and incubated at 37°C in the dark for 40 min. The mixture of 2 ml distilled water and 0.2 ml DPPH solution served as a control (A_control_). A blank sample was prepared by replacing the DPPH solution with ethanol (A_blank_). The absorbance of the sample after incubation was measured at 517 nm using a UV‐1600 spectrophotometer (A_sample_). Lower the measurement of A_sample_, stronger the scavenging ability of DPPH. The percentage of DPPH‐scavenging (DPPH_scav_) activity was calculated as follows: Equation (1):

(1) $${\text{DPPH}}_{\text{scav}}\left(\%\right)=\left(1‐\frac{A_{\text{sample}}‐A_{\text{sample control}}}{A_{\text{blank}}}\right)\times 100$$

#### 2.4.2 Hydroxyl radical scavenging activity measurement

The hydroxyl radical scavenging activity of the samples was measured according to the work published by Herraiz and Galisteo (2015) with slight modification. A total of 100 μl of the redissolved solution was sequentially mixed with 250 μl of 100 mM phosphate buffer solution (pH 7.4), 25 μl of 10 mM ferrous sulfate solution, 25 μl of 10 mM EDTA solution, and 25 μl of 10 mM α‐deoxyribose solution. Next, 50 μl of 10 mM hydrogen peroxide solution was added, followed by shaking of the mixture for 30 s, allowing to stand at 37°C for 15 min before adding 250 μl of 2.8% trichloroacetic acid and 250 μl of 1% TBA solution, and mixing thoroughly, and then measuring the absorbance at 325 nm (A_sample_). Distilled water served as the blank (A_blank_). Solutions of ferrous sulfate were made immediately before use. Lower the measurement of A_sample_, stronger the hydroxyl radical scavenging activity. The scavenging activity (%) was calculated according to the following Equation (2):

(2) $$\text{Hydroxyl radical scavenging activity (}\%)=\left(\frac{A_{\text{blank}}‐A_{\text{sample}}}{A_{\text{blank}}}\right)\times 100$$

#### 2.4.3 Lipid peroxidation inhibition measurement

The lipid peroxidation inhibition capacity was determined according to the work published by Yang and Stockwell (2016) with slight modifications. A total of 200 ml of the redissolved solution was mixed with 1 g of peanut oil and a 5 ml solution containing thiobarbituric acid (15%, w/v), thrichloracetic acid (0.37%, w/v), and hydrochloric acid (1.8%, v/v). The mixture was heated at 90°C for 6 h in a water bath to promote the formation of a pink pigment. Afterwards, the mixture was cooled rapidly in an ice bath, centrifuged for 5 min at 2000 rpm/min, and filtrated. The absorbance of the filtrate was measured by a spectrophotometer at 532 nm (A_sample_). A blank was prepared by replacing the sample with distilled water (A_blank_). Lower the measurement of A_sample_, stronger the lipid peroxidation inhibition capacity. The lipid peroxidation inhibition capacity (%) was calculated according to the following Equation (3):

(3) $$\text{Lipid peroxidation inhibition capacity (}\%)=\left(\frac{A_{\text{blank}}‐A_{\text{sample}}}{A_{\text{blank}}}\right)\times 100$$

### 2.5 Gas chromatography–mass spectrometry (GC‐MS) analysis

The polyphenol complexes extracted from the three seaweed species under the optimized processing condition of each were subjected to gas chromatography–mass spectrometry (GC‐MS) analyses (Rahaman et al., 2019, 2020). GC‐MS analyses were performed on a Thermo Fisher (San Jose, CA) TRACE DSQ single‐quadrupole mass spectrometer following the method developed by Verslues (2017) with slight modifications. The GC conditions were as follows: column, ZB‐5MS (Phenomenex; Torrance CA), 30 m × 0.25 mm, 0.25 µm film thickness; carrier gas, helium; linear velocity, 1.3 ml/min (constant flow); split flow, 10 ml/min; injector temperature, 230°C; column temperature program, and initial temperature of 40°C held for 1 min followed by an increase to 310°C at 5°C/min. The MS conditions were as follows: ionization, electron impact (70 eV); detection, positive ion; full‐scan analyses, 10 m/z–600 m/z at 2 scans/s. Volatile metabolites were eluted with the solvent front using this method, so GC separation of these analyses started with an initial temperature of 40°C held for 2 min, followed by an increase to 80°C at 10°C/min. The temperature was maintained for 3 min at 80°C after which it was increased to 230°C at a rate of 30°C/min.

### 2.6 Data analysis

Measurements were performed in triplicate. Data were presented as the mean with standard deviation and subjected to one‐way analysis of variance (ANOVA) and least significant difference (LSD) using MINITAB Statistical Software v15. The significance was judged statistically by the *F* value at probability (*p*) below .05.

## 3 RESULTS AND DISCUSSION

### 3.1 Process optimization

Table 2 shows the total polyphenolic content of the permeate after filtration and trial of the collected species according to the orthogonal design. The R value demonstrates the importance of the factor to the process. Judged by the R value of the different seaweed species, *Sargassum miyabei, Undaria pinnatifida suringar*, and *Sargassum thunbergii*, the most influential factors were the ethanol concentration (R = 0.5200), ethanol concentration (R = 0.8000), and processing temperature (R = 1.1900), respectively. The least influential factors were the ratio of powder to liquid (R = 0.23), processing time (R = 0.08), and the ratio of powder to liquid (R = 0.23), respectively. The higher *K* value of each column indicated stronger impact. This demonstrated that the optimized processing conditions for the different seaweed species of *Sargassum miyabei, Undaria pinnatifida suringar,* and *Sargassum thunbergii* were A_2_B_1_C_3_D_1_ (processing temperature of 60°C, ethanol concentration of 70%, ratio of powder to liquid of 1:10, and processing time of 3 h); A_3_B_1_C_1_D_2_ (processing temperature of 70°C, ethanol concentration of 70%, ratio of powder to liquid of 1:8, and processing time of 4 h), and A_3_B_1_C_1_D_2_ (processing temperature of 7°C, ethanol concentration of 70%, ratio of powder to liquid of 1:8, and processing time of 4 h), respectively. The total polyphenol content produced from the optimized conditions of each seaweed species was compared with that from the nine trials. It was found that for each seaweed species, the total polyphenol content of the permeate after filtration produced by optimized processing conditions significantly exceeded from each of the nine trials of the same species. Among others, the total polyphenol content produced by the optimized processing conditions from *Sargassum thunbergii* was significantly higher than the others.

### 3.2 Antioxidative activity of polyphenols produced from optimized processing conditions of *Sargassum miyabei*, *Undaria Pinnatifida Suringar*, and *Sargassum thunbergii*

The antioxidative activity of hydrolysates has been reported in polyphenols extracted from different food sources such as almond (Bolling, 2017), black tea (Tenore et al., 2015), and algae (Machu et al., 2015). Our study found that among the polyphenols extracted from the three species of *Sargassum miyabei, Undaria pinnatifida suringar*, and *Sargassum thunbergii* under the optimized processing conditions, *Undaria pinnatifida suringar* demonstrated the strongest antioxidative activity. The three methods of DPPH scavenging ability, hydroxyl radical scavenging activity, and lipid peroxidation resistance ability were used to measure antioxidative activity. Although these different methods were based on different principles, all three sets of results demonstrated the same order: the antioxidative activity of polyphenols extracted from *Undaria pinnatifida suringar* was the strongest among the three, while the antioxidative activity of polyphenols extracted from *Sargassum thunbergii* was the weakest. For example, regarding the DPPH radical scavenging activity measurement, the polyphenols extracted from *Undaria pinnatifida suringar* and *Sargassum thunbergii* demonstrated the highest value of 92.95%, and the lowest value of 39.31%, respectively. The trend of these three sets of results complements each other.

### 3.3 GC‐MS analysis of polyphenols produced from different seaweed species

**FIGURE 1:**
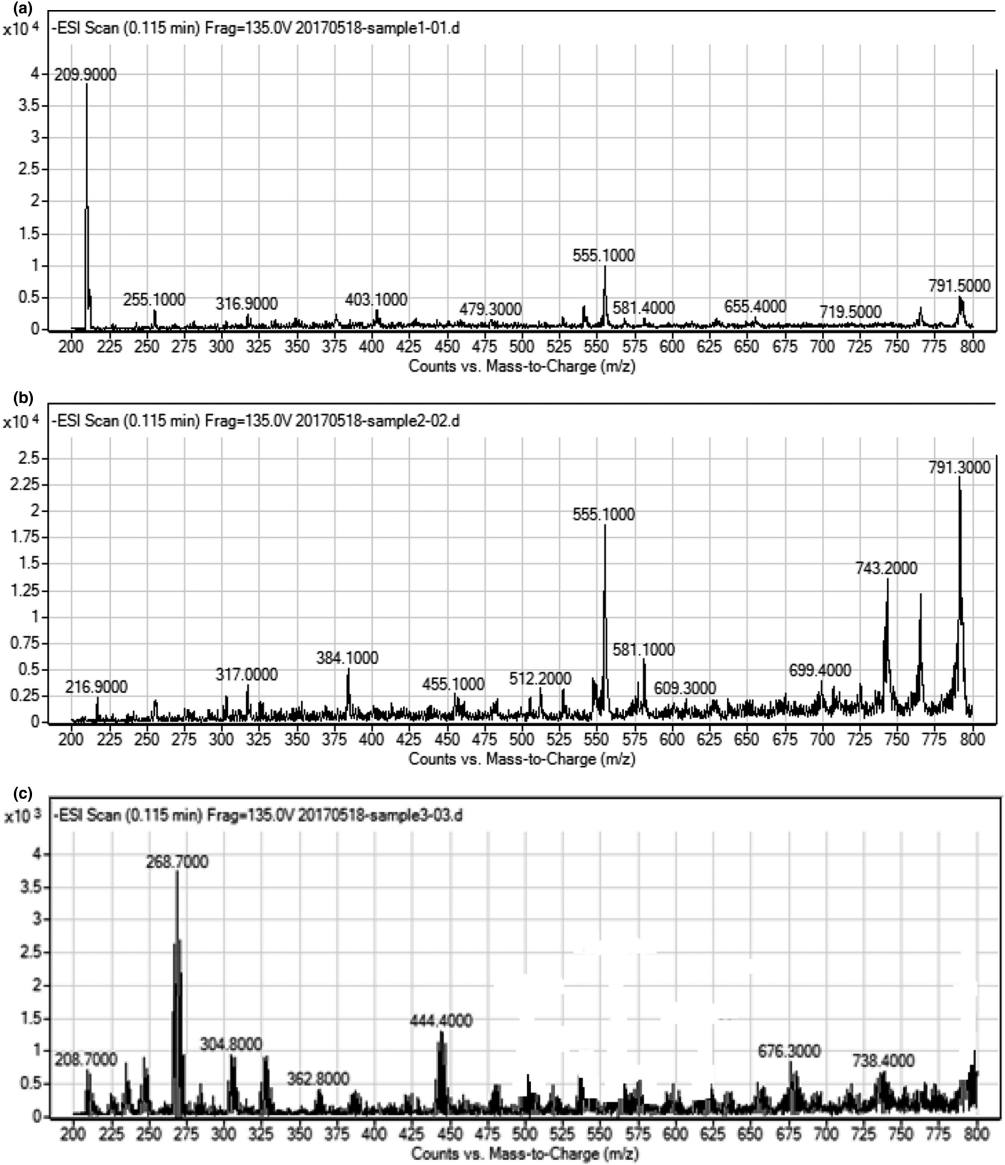
Gas chromatography–mass spectrometry (GC‐MS) analysis of the polyphenols extracted from the different seaweed species: (a) *Sargassum miyabei*; (b) *Undaria pinnatifida suringar*; and (c) *Sargassum thunbergii*

The different antioxidative activity of the polyphenol complexes extracted from the three seaweed species indicated the different components of the polyphenols. The GC‐MS analysis was carried out to investigate these different components of polyphenols (Figure 1). By comparing the peaks in the diagrams of *Sargassum miyabei*, *Undaria pinnatifida suringar,* and *Sargassum thunbergii*, the two outstanding peaks with the most intensity appeared at 555.1 m/z and 791.3 m/z (Figure 1b), and the most outstanding peak appeared at 209.9 m/z and 268.7 m/z (as shown in Figure 1a,c), respectively. These peaks were identified by NIST14.L library retrieval analysis. The result showed that these peaks represented the components of syringate (209.9 m/z, Figure 1a), gallic acid (555.1 m/z, Figure 1b), arbutin (791.3 m/z, Figure 1b), and phloretin (268.7 m/z, Figure 1c). The antioxidative activity of gallic acid and arbutin has been reported broadly. Badhani et al. (2015) reviewed the gallic acid as a versatile antioxidant with promising therapeutic and industrial applications. Roidoung et al. (2016) used gallic acid as a protective antioxidant against anthocyanin degradation and color loss in vitamin C fortified cranberry juice. They concluded that gallic acid was able to preserve health beneficial components and the endogenous red color in cranberry juice after 16 days of storage time. However, the control sample without gallic acid did not show these characteristics. Fatemeh et al. (2015) found that the antioxidant capacity of 50 mg/kg/bw arbutin had a protective effect on lipid peroxidation and cyclosporine‐induced toxicity. Furthermore, Erenler et al. (2016) reported that the compounds extracted from *Origanum majorana* exhibited significant antioxidant activities. They stated that the arbutin was one of the major compounds in the sample. Furthermore, DPPH free radical scavenging assay proved that the antioxidative activity of arbutin (45%) was the highest followed by butylated hydroxytoluene (15%). However, the antioxidative activity of syringate (Figure 1a) and phloretin (Figure 1c) has rarely been reported.

## 4 CONCLUSIONS

Polyphenol complexes were extracted from the three major seaweed species (*Sargassum miyabei, Undaria pinnatifida suringar*, and *Sargassum thunbergii*) of Guangdong Province, the coastal province with the longest coastline in China. The extraction condition of each seaweed species was optimized according to the orthogonal method. The polyphenol complexes extracted from each seaweed species were further subjected to antioxidative measurement by three methods: the DPPH radical scavenging acidity measurement, hydroxyl radical scavenging activity measurement, and liquid peroxidation inhibition measurement. All three methods confirmed that the polyphenol complex extracted from *Undaria pinnatifida suringar* demonstrated the highest antioxidative activity. Gas chromatography–mass spectrometry analysis revealed the high content of gallic acid and arbutin in the polyphenols extracted from *Undaria pinnatifida suringar*, whereas the highest contents extracted from *Sargassum miyabei* and *Sargassum thunbergii* were syringate and phloretin, respectively. However, the antioxidant effect of syringate and phloretin has rarely been reported.

## CONFLICT OF INTEREST

All authors declare no conflicting interests.
